# Supplementary material for: Eater cooperates with Multiplexin to drive the formation of hematopoietic compartments
Source: eLife. 2020 Oct 7;9:e57297. doi: 10.7554/eLife.57297 (PMC7541089; doi:10.7554/eLife.57297)
Supplement: Transparent reporting form [file elife-57297-transrepform.docx]

***eLife’s* transparent reporting form**

We encourage authors to provide detailed information *within their submission* to facilitate the interpretation and replication of experiments. Authors can upload supporting documentation to indicate the use of appropriate reporting guidelines for health-related research (see [EQUATOR Network](http://www.equator-network.org/%20)), life science research (see the [BioSharing Information Resource](https://biosharing.org/)), or the [ARRIVE guidelines](http://www.plosbiology.org/article/info:doi/10.1371/journal.pbio.1000412) for reporting work involving animal research. Where applicable, authors should refer to any relevant reporting standards documents in this form.

If you have any questions, please consult our Journal Policies and/or contact us: [editorial@elifesciences.org](mailto:editorial@elifesciences.org).

**Sample-size estimation**

- You should state whether an appropriate sample size was computed when the study was being designed
- You should state the statistical method of sample size computation and any required assumptions
- If no explicit power analysis was used, you should describe how you decided what sample (replicate) size (number) to use

Please outline where this information can be found within the submission (e.g., sections or figure legends), or explain why this information doesn’t apply to your submission:

The description can be found in the **Materials and Methods** section under the "**Cell quantification and statistical analysis**" heading:

Sample-size criteria was estimated *post hoc* with G*Power 3.1 (Faul et al., 2009). All significantly different datasets exceeded 0.99 Power (1-beta) with respective sample sizes, means and standard deviations.

**Replicates**

- You should report how often each experiment was performed
- You should include a definition of biological versus technical replication
- The data obtained should be provided and sufficient information should be provided to indicate the number of independent biological and/or technical replicates
- If you encountered any outliers, you should describe how these were handled
- Criteria for exclusion/inclusion of data should be clearly stated
- High-throughput sequence data should be uploaded before submission, with a private link for reviewers provided (these are available from both GEO and ArrayExpress)

Please outline where this information can be found within the submission (e.g., sections or figure legends), or explain why this information doesn’t apply to your submission:

All replicates used for statistical analyses in the manuscript are shown in **source data files** corresponding to the relevant experiments. Replicate numbers are also indicated in the **Figure legends.**

**Statistical reporting**

- Statistical analysis methods should be described and justified
- Raw data should be presented in figures whenever informative to do so (typically when N per group is less than 10)
- For each experiment, you should identify the statistical tests used, exact values of N, definitions of center, methods of multiple test correction, and dispersion and precision measures (e.g., mean, median, SD, SEM, confidence intervals; and, for the major substantive results, a measure of effect size (e.g., Pearson's r, Cohen's d)
- Report exact p-values wherever possible alongside the summary statistics and 95% confidence intervals. These should be reported for all key questions and not only when the p-value is less than 0.05.

Please outline where this information can be found within the submission (e.g., sections or figure legends), or explain why this information doesn’t apply to your submission:

**Figure 1E - Figure 1-source data 1** (description of statistical test used, p-values, and error bars are found in the figure legend)

**Figure 1-figure supplement 1C -** **Figure 1-source data 2**(description of statistical test used, p-values, and error bars are found in the figure legend)

**Figure 2I -** **Figure 2-source data 1** (description of statistical test used, p-values, and error bars are found in the figure legend)

**Figure 2J -** **Figure 2-source data 2** (description of statistical test used, p-values, and error bars are found in the figure legend)

**Figure 3K -** **Figure 3-source data 1** (description of statistical test used, p-values, and error bars are found in the figure legend)

**Figure 3O - Figure 3-source data 2** (description of statistical test used, p-values, and error bars are found in the figure legend)

**Figure 3-figure supplement 1I -** **Figure 3-source data 3** (description of statistical test used, p-values, and error bars are found in the figure legend)

**Figure 3-figure supplement 2H -** **Figure 3-source data 4** (description of statistical test used, p-values, and error bars are found in the figure legend)

**Figure 4E -** **Figure 4-source data 1** (description of statistical test used, p-values, and error bars are found in the figure legend)

**Figure 4J -** **Figure 4-source data 2** (description of statistical test used, p-values, and error bars are found in the figure legend)

**Figure 4M -** **Figure 4-source data 3** (description of statistical test used, p-values, and error bars are found in the figure legend)

**Figure 5E -** **Figure 5-source data 1** (description of statistical test used, p-values, and error bars are found in the figure legend)

**Figure 5F -** **Figure 5-source data 2** (description of statistical test used, p-values, and error bars are found in the figure legend)

Raw data and exact *n*-numbers are shown in **the indicated Source data files**

(For large datasets, or papers with a very large number of statistical tests, you may upload a single table file with tests, Ns, etc., with reference to sections in the manuscript.)

**Group allocation**

- Indicate how samples were allocated into experimental groups (in the case of clinical studies, please specify allocation to treatment method); if randomization was used, please also state if restricted randomization was applied
- Indicate if masking was used during group allocation, data collection and/or data analysis

Please outline where this information can be found within the submission (e.g., sections or figure legends), or explain why this information doesn’t apply to your submission:

Groups were allocated according to corresponding genotypes or experimental conditions. No masking was applied.

**Additional data files (“source data”)**

- We encourage you to upload relevant additional data files, such as numerical data that are represented as a graph in a figure, or as a summary table
- Where provided, these should be in the most useful format, and they can be uploaded as “Source data” files linked to a main figure or table
- Include model definition files including the full list of parameters used
- Include code used for data analysis (e.g., R, MatLab)
- Avoid stating that data files are “available upon request”

Please indicate the figures or tables for which source data files have been provided:

**Figure 1E - Figure 1-source data 1**: Quantification of FBAH number in control and *C7>Atf3^WT^* larvae.

**Figure 1-figure supplement 1C -** **Figure 1-source data 2**: Circulating hemocyte counts from control and Atf3 overexpressing larvae. For graphical representation, the data was normalized to the average of the control.

**Figure 2I -** **Figure 2-source data 1:** Quantification of FBAH number in *C7>Atf3^WT^* larvae without parasitoid wasp infection and 48 hours post-infection.

**Figure 2J -** **Figure 2-source data 2:** The percentage of lamellocytes 24 hours after *L. boulardi* infestation in control and *C7>Atf3^WT^* larvae.

**Figure 3K -** **Figure 3-source data 1:** Quantification of FBAH number in *C7>Atf3^WT^* and *C7>Atf3^WT^Mp^RNAi^* larvae.

**Figure 3O - Figure 3-source data 2**: Quantification of sessile hemocyte intensity in control, *C7>Atf3^WT^* and *C7>Atf3^WT^Mp^RNAi^* larvae. For graphical representation, the data was normalized to the average of the control.

**Figure 3-figure supplement 1I -** **Figure 3-source data 3**: Quantification of FBAH number in *C7>Atf3^WT^, C7>Atf3^WT^Col4a1^RNAi^, C7>Atf3^WT^trol^RNAi^, C7>Atf3^WT^vkg^RNAi^, C7>Atf3^WT^LanA^RNAi^, C7>Atf3^WT^LanB1^RNAi^* and *C7>Atf3^WT^LanB2^RNAi^* larvae.

**Figure 3-figure supplement 2H -** **Figure 3-source data 4**: Quantification of FBAH number in Control, *C7>Atf3^WT^, C7>Atf3^WT^Mp^RNAi^, C7>Atf3^WT^SPARC^RNAi^* and *C7>Atf3^WT^SPARC^RNAi^Mp^RNAi^* larvae.

**Figure 4E -** **Figure 4-source data 1**: Quantification of FBAH number in control, *C7>Atf3^WT^* and *C7>Atf3^WT^eater^1^* larvae.

**Figure 4J -** **Figure 4-source data 2**: Quantification of FBAH number in *C7>Hml>Atf3^WT^* and *C7>Hml>Atf3^WT^eater^RNAi^* larvae.

**Figure 4M -** **Figure 4-source data 3**: Quantification of sessile hemocyte intensity in control and *a58>Mp^RNAi^* larvae. For graphical representation, the data was normalized to the average of the control.

**Figure 5E -** **Figure 5-source data 1**: Quantification of sessile hemocyte intensity in control and *C7>Mp::GFP* larvae. For graphical representation, the data was normalized to the average of the control.

**Figure 5F -** **Figure 5-source data 2**: Circulating hemocyte counts from control, Mp::GFP overexpressing and *eater^1^* mutant larvae. For graphical representation, the data was normalized to the average of the control.
